# Some of the Evils That Threaten the Medical Profession*Read before the Richmond Academy of Medicine and Surgery. May 11th, 1897.

**Published:** 1897-06

**Authors:** W. S. Beazely

**Affiliations:** Richmond, Va.; Demonstrator of Physiology, Medical College of Virginia, etc.; 2600 East Broad Street


### SOME OF THE EVILS THAT THREATEN THE MEDICAL PROFESSION.*

By W. S. BEAZELY, M. D., Richmond, Va.

Demonstrator of Physiology, Medical College of Virginia, etc.

A few meetings ago, Dr. Hodges, in his most excellent paper, on some of the socio-medical problems which confronted us as professional men, aroused the members of the Academy to action. I wish to add to what he has already said.

In these days, when competition is so great, men are offering their services almost without money and without price. The same competition has found its way into the medical profession, and we find its followers offering their services for a small consideration.

Before mentioning some of the evils which threaten to bring our profession into disrepute, I will make two assertions which can hardly be contradicted: *First.*—There is no class of men in a position to be as independent as the men of our profession. *Second.*—No class of men do more charity work than do physicians.

My first proposition that physicians can be the most independent of all men, will appear conclusive if we consider the appeals made to us by the maimed, the halt, and the blind. Medical attention is a necessity, and who can render these services save those who have unlocked the mysteries of the human system and studied diseases contained therein. People may, and often do without treatment of a sin-sick soul; but when suffering flesh is made to cry out in agony, then the medical man is sought for relief, and he is expected to respond, even though he fails to receive money or thanks for his services.

Notwithstanding this age of educational advantages, scientific research and progress in lines of business, we may pause, survey the field, and ask the question, whether or not our profession is advancing, or whether we are being brought into disrepute?

In order to answer such questions, there are certain evils to be considered. The first to which I will call attention is the spirit of jealousy existing among men who should be the foremost in the defense of ethics. This evil has invaded our city, and entered the hearts of many of us. Jealousy and uniform

---

*Read before the Richmond Academy of Medicine and Surgery. May 11th, 1897.

ity of action are incompatible. With this fact staring us in the face, what is to become of the true worth of our calling?

With uniformity of action, our profession would not only occupy a high place in the estimation of the laity, but the practice of it would be worth to the physicians of Richmond several times as much financially as it is now. How often do we answer calls to families only to find out that a dozen or more physicians have given them up because they do not pay. How often, in the still hour of the night, do we arise, shake off the hold of "nature's sweet restorer," to relieve a patient, and hear later, through some of the neighbors, that he doesn't pay doctors' bills? Under the present system of things, some people rarely if ever pay a doctor's bill, for where "patience ceases to be a virtue," and the doctor urges his claim, he is discharged and another called in. Of course, those whose hearts are willing, but whose flesh is weak, are not included in the list of those who delight in seeing our mental faculties burn with anxiety for their suffering flesh without compensation.

It is a sad fact that some people are not even grateful for services rendered. This may be accounted for by the fact that gratitude, being a debt, receives the same treatment as all other debts.

The obligations of patient and physician are mutual, and the physician has the right to expect from his patient the consideration of his health and comfort, and the prompt payment for services rendered.

We will all agree that in proportion to the amount of work we are more poorly paid than any other class of people.

That there is a cause for this state of affairs, all will agree. That we are responsible for the trouble, some will question. Let us see how far we are responsible. Mr. A has his druggist to 'phone for Dr. B, and Dr. B refuses because he has not been paid for previous visits. He then calls upon a physician who does not know him, but who, after a long service of faithful work, knows him to his sorrow. When the second physician becomes disgusted and quits, another one is likewise imposed upon, and so on down the list, until the man has lived a lifetime without paying a doctor's bill. A certain man was known to make the brag to a collector that he didn't pay doctor's bills. He sent for me, thinking that I was innocent of his fraudulent methods. I refused to attend him, whereupon he secured another physician. The last I heard of him he was changing physicians, and the Lord only knows when he will stop. I might enumerate other cases, but all of us know too well that such a state of affairs exist.

Now, if we agree that we can better the condition of affairs, we must also agree that we are largely responsible for non-appreciation and non-payment of bills by a large number of people.

What is the matter? I might sum it up in these words: *Lack of Unity.* Until we realize that we are one in the profession of medicine, and defenders of each other, there will be discord in the ranks. The dead-beats we have with us, but all of us don't know them. They should be introduced to every physician in Richmond. Let them be known to all our medical brethren, and practice will be worth double as much as it is. In this day, when a great financial crisis is upon us, some of us look, with hands off, at questions of common interest, for fear of losing a few dollars. Selfishness makes us narrow, and we fail to accomplish the purpose for which we were intended.

There is another evil which is threatening our profession, and that is the prescribing of patent and proprietary remedies. I wish to submit three propositions, and try to prove them to you:

1st. The prescribing of such remedies is unscientific.

2d. It is causing the general public to prescribe for themselves.

3d. It is increasing the number of manufacturing chemists, and thereby allowing them the privilege of dictating to us as to treatment of disease.

To prescribe a remedy about which we know nothing is unscientific and unsatisfactory to the physician. All of the patent medicines, and many of the proprietary remedies, are secret preparations. I will mention three remedies, which will illustrate my point. These are ammonal, pyroctin, and acetifas. If the formula for ammonal has been given, I have never seen it. Pyroctin is said to be a febrifuge, anodyne, and antipyretic. What is the nature of pyroctin? Does it grow on trees, or come out of the ground? We are told that acetifas is acetifas and citrate of caffein. After we have read the formula, we may pause and ask: What is acetifas? We teach our men at college to formulate their own prescriptions. Let us practice what we preach.

I have been told that some of our medical brethren have looked with favor upon Castoria, while a few have given Mrs. Winslow's Soothing Syrup consideration.

In regard to the second point, that it teaches the laity to prescribe for themselves, we have only to listen to our patients as they tell us Dr. So-and-So prescribes Henry's tri-iodides and chlorides. Some people buy these ready-prepared mixtures rather than send for a physician, because of the fact of their hearing that physicians prescribe them.

As to the third point, that such prescribing is increasing the number of manufacturing chemists, and thereby allowing them to dictate to us as to treatment of disease—we have only to look on our tables, book-cases, and mantelpieces, and behold a multitude of worthless remedies. Representatives of these various remedies tell us all about them, the indications for their use, and the surety of their action. Sometimes we take their advice.

Our position should be that of leaders, and not followers. We should be the teachers in this medical world, and elevate rather than lower our profession. We may go back to the days of the pioneers of our noble calling, and ask ourselves if we hold the honor and the dignity of our profession as sacred as they did?

Another evil found among us is our willingness to give testimonials. We not only prescribe patent and proprietary remedies, but we give testimonials as to their merits. These testimonials often go before the public, and, as a result, they patronize these remedies rather than the doctor. May the time soon come when physicians will say to the manufacturers, "We are tired of having you tell us what we want and ought to use in our practice. We will prescribe what we need and get our local druggists to fill the prescriptions."

Of course, there are preparations whose formulas are given, and which have proven beneficial in the treatment of disease. Cruel, indeed, would we be to withhold the means of alleviating the pangs of human suffering; but in treating our patients, let us know what we are giving.

As physicians, we ought to be practical men. In administering remedies, we should have a definite purpose in view, and use those from which, from our knowledge of the physiological action of drugs, we can expect results.

To experiment with the secret combinations of remedies now being sent out to the physicians and the public, is unwise.

The man who fires a load of shot into a covey of birds, hoping that some of them may get a bird, does not deserve as much credit as the man who, taking aim at one particular bird, brings him down.

And now, Mr. President, in closing let me urge the members of this Academy to defend not only their individual interests, but the great principles upon which rests our profession. Narrow, indeed, is the world to the man whose ideas are confined to the building up of himself; but he whose life is thrown into the life of others lives in closer touch with the spirit of progress which has blessed our nation.

Some may say to young men, "Our profession is overcrowded; stay out." I would answer them by saying there is room for good men in every calling of life; come on. Let us push onward and upward in the medical world and always remember that it is a good thing for "brethren to dwell together in unity."

*2600 East Broad Street.*

